# COVID-19 Related Traumatic Distress in Psychotherapy Patients during the Pandemic: The Role of Attachment, Working Alliance, and Therapeutic Agency

**DOI:** 10.3390/brainsci11101288

**Published:** 2021-09-28

**Authors:** Katie Aafjes-van Doorn, Vera Békés, Xiaochen Luo

**Affiliations:** 1 Ferkauf Graduate School of Psychology, Yeshiva University, Rousso Building, 1165 Morris Park Avenue Bronx, New York, NY 10461, USA; vera.bekes@yu.edu; 2 Department of Counseling Psychology, Santa Clara University, Santa Clara, CA 95053, USA; xluo@scu.edu

**Keywords:** COVID-19, psychotherapy, online, attachment, alliance, agency

## Abstract

The early months of the COVID-19 pandemic have been a challenging time for many psychotherapy patients. To understand why certain patients were more resilient, we examined the role of patients’ attachment anxiety and attachment avoidance, as well as collaborative therapy experiences (perceived working alliance and therapeutic agency) in their online sessions on their COVID-related traumatic distress over a three-month period. A total of 466 patients in online psychotherapy completed a survey during the first weeks of the pandemic, and 121 of those completed a follow-up survey three months later. Lower distress at follow-up was predicted by patients’ lower attachment anxiety and higher therapeutic agency in their online sessions after controlling for baseline distress and time of survey completion. Higher working alliance predicted less distress at follow-up only for patients with high attachment anxiety. For patients with low attachment avoidance (i.e., more securely attached), higher therapeutic agency predicted less distress. These findings suggest that patients’ attachment anxiety and therapeutic agency may play significant roles also in online therapy during COVID-19 in patient’s experienced traumatic distress, and that working alliance and therapeutic agency may be differentially important for patients with different levels of attachment anxiety and avoidance.

## 1. Introduction

The rapid spread and devastating impact of COVID-19 has led governments’ to impose global social distancing measures (i.e., public health measures taken to restrict when and where people can gather), lockdowns, quarantine requirements, and curfews in order to slow the spread of the virus. The pandemic has had ripple effects on the daily lives of many, putting into question our basic assumptions about medical and financial safety and stability in our lives. It is therefore not surprising that the pandemic has shown to adversely affect our well-being, and increase psychological distress [1,2,3,4]. Many individuals reported a fear of COVID-19 [5], which has been linked to increased levels of depression and anxiety [6,7]. 

Arguably, the pandemic has been especially stressful for individuals with pre-existing mental health problems. Patients with diagnosed mental health disorders have reported not only more personal worries about COVID-19 and fear of contagion than healthy controls, they have also reported increased psychosocial distress [8] and worsening of their symptomatology [9,10,11], increasing their need for psychological support. 

For those individuals who were in psychotherapy, additional challenges emerged as therapy sessions could not continue in-person due to the social restrictions imposed by health authorities. Millions of in-person therapies transitioned to online therapy via videoconferencing at once, without much preparation or support [12]. With the ongoing pandemic and its ripple effects on individuals’ lives, attending online psychotherapy may provide support for patients in managing the experienced COVID-related distress. However, the therapy process might not be equally important for all patients; there might be differences in the utilization of the online therapy process between patients with different levels of attachment anxiety and avoidance.

### 1.1. The Importance of Patients’ Attachment in Psychological Distress

Bowlby’s attachment theory posits that infants develop a deep and enduring emotional bond with their primary caregivers in which they seek closeness and feel more secure, and that individual differences in these early relationships are carried forward and shape relationships with others in adulthood [13,14,15,16,17,18]. Attachment representations tend to be stable over time [19], representing a patient-trait rather than in-session state, and can be categorized based on two dimensions, anxiety and avoidance [20,21]. Attachment anxiety involves a fear of rejection or abandonment by others, excessive need for approval, and distress when others are unavailable or unresponsive. On the other hand, attachment avoidance involves fear of intimacy and dependence, excessive need for self-reliance and reluctance of self-disclosure. Individuals who endorse high levels of either attachment anxiety or attachment avoidance or both, are deemed to have an insecure attachment, whereas low scores on both attachment anxiety and attachment avoidance indicate a secure attachment [22]. Securely attached individuals tend to rely effectively but not exclusively on others and enjoy reciprocal and collaborative relationships [23]. Both attachment anxiety and avoidance are seen as a continuum on which all individuals can be placed, endorsing more or less of these insecure attachment representations in their interpersonal relationships. Although dimensional models of attachment are argued to be better suited for conceptualizing and measuring individual differences in attachment representations than simple categories of secure/insecure or avoidant/anxious [24], many research studies have reported on patients’ attachment security or lack of security to understand the development and treatment of various psychological problems and psychopathologies [25,26,27,28]. Studies suggest that patients in clinical samples more often report an insecure attachment, compared with non-clinical samples [29], for a recent meta-analysis, see [30]. Similarly, compared to securely attached individuals, patients with an insecure attachment tend to experience higher levels of symptoms [31,32,33], including PTSD symptoms [34], and tend to do worse in psychotherapy [23].

With regards to post-traumatic stress, there is debate within the literature about the role of avoidance in the onset and perseverance of PTSD symptoms, for a summary see [35]. A large meta-analysis on the relationship between attachment and PTSD symptoms indicated that attachment avoidance does not seem to be related to symptoms of PTSD, whereas high levels of attachment anxiety have shown to be strongly related to PTSD symptoms [34]. It has been suggested that avoidance of threat-related cues, thoughts and feelings, combined with avoidance of attachment related worries, may be beneficial when recovering from a traumatic event [36]. Others have highlighted that in patients with high attachment anxiety, their intense dependency needs may make their use of interpersonal relationships more imminent, especially during times of traumatic distress. In therapy, for example, patients’ attachment anxiety may put a high demand on the therapeutic relationship and on the therapist, sometimes leading to poorer outcomes [23].

Psychotherapy treatment outcomes also appear to differ based on patients’ level of attachment anxiety and avoidance [37]. Generally, securely attached patients have the best psychotherapy outcomes, better than those with high attachment avoidance, and/or high attachment anxiety. Some evidence suggests that individuals with high attachment avoidance are doing slightly better in therapy than those high on attachment anxiety, but are also more likely to drop out of treatment prematurely [32,37]. Interestingly, a recent empirical study indicated that patients’ level of distress during the course of in-person psychotherapy treatment was related to their level of attachment anxiety but not their level of attachment avoidance [38].

### 1.2. Patients’ Experiences of the Collaborative Process in Therapy

The concept of attachment has been intimately linked to the in-session therapy process, in fact, Bowlby (1988) [13] suggested that early attachment representations get reactivated in therapy with the therapist, who fulfills similar functions as the patients’ early attachment figures [13]. In other words, the therapist and the therapeutic context function as a secure base where the patient can (re-) experience internalized interpersonal attachment patterns within an attuned therapeutic relationship. At its best, the therapy offers the patient a new attachment experience with the therapist that is imbued with meaning and empathic connection, that contributes to a positive therapy process and treatment outcome, e.g., see [39]. Two in-session therapy processes might be particularly relevant to the patients’ attachment; the patients’ perception of the quality of the working alliance and their sense of therapeutic agency in the session itself.

The working alliance, the relationship between therapist and patient, in which both parties strive to work together and achieve positive change for the patient, is perhaps the most important and powerful factor when it comes to making progress in therapy [40]. Despite therapists’ concerns about the ability to develop a strong working alliance in online settings [41,42], research suggests that the working alliance in online psychotherapy is usually strong [43] and comparable to in-person therapies [44,45]. Although there is lack of research regarding the impact of the working alliance on online psychotherapy, the working alliance has been suggested to be crucial for good outcomes in online therapy via videoconferencing [46]. With regards to patients’ attachments, individuals with a more secure attachment tend to report a stronger working alliance with their therapist, and more insecurely attached patients report a lower working alliance (for a meta-analysis, see [47]). More specifically, both attachment avoidance and attachment anxiety have shown to be negatively correlated with reports of the working alliance in-session, for a meta-analysis, see [48]. 

Besides patients’ attachment and the working alliance, patients’ sense of agency, reflecting a capacity for self-direction and the degree to which one believes they have control over their life, has also shown to be a predictor of lower distress and better treatment outcome [49,50]. Individuals’ sense of agency is thought to influence how people understand their interpersonal relationships, and is an important contributor to behavioral regulation, and promotion and maintenance of interpersonal functioning [51]. Recent empirical studies have linked patient’s sense of agency to their attachment representations, in that individuals with a greater sense of agency tend to be more securely attached [52], and those with lower agency tend to report more attachment anxiety. Across psychotherapeutic traditions, agency is an important indicator of positive psychological functioning, in that therapists tend to view patients as active agents, who use the therapists’ support and interventions to heal themselves [53]. It is thought that a low sense of agency may exacerbate relationship difficulties, also within the therapist-patient dyad [52]. Patients’ sense of agency may manifest within psychotherapy sessions as their contribution to the therapy process, openness, expressiveness, cooperativeness, autonomy, contribution to the therapeutic bond, active collaboration, and interactive collaboration with the therapist [54], and has been associated with a stronger working alliance [49,55,56]. 

This intentional influence over the psychotherapy change (i.e., ‘therapeutic agency’) has been proposed as the single most important determinant of therapy outcome [54]. Recently, several empirical studies have indeed found therapeutic agency to be associated with the working alliance [49,56], lower psychological distress, lower depression levels, and better therapy outcomes even when controlling for baseline distress [57]. Moreover, increases in therapeutic agency over the course of psychotherapy have shown to predict subsequent symptom improvement [56]. 

### 1.3. Patient’s Attachment as a Moderator of the Process-Outcome Associations

Despite the importance of in-session collaborative experiences such as the working alliance or therapeutic agency on treatment outcomes, their impacts may vary depending on patients’ attachment styles. Indeed, previous studies in adolescent samples suggested that a strong working alliance may be especially helpful for patients with insecure attachment, whereas the alliance-outcome relationship was attenuated when patients reported a secure attachment history, e.g., [58]. This indicates that enhancing collaborative therapeutic experiences may have a buffering effect especially for patients with less secure attachment representations.

Based on the interpersonal differences between individuals who have high attachment anxiety or avoidance, further differences may be expected. For example, for patients with high levels of attachment anxiety, it might be crucial to experience a strong collaborative relationship with their therapist to be able to address the patient’s dependency needs, before they are able to fully engage in the interventions and make progress [23] In contrast, for patients with high attachment avoidance, the patient’s need for independence may translate into less demand on the collaborative process itself in achieving improvement over the course of therapy.

In this study we aim to investigate whether, during the initial phase of the ongoing COVID-19 pandemic, patients’ attachment anxiety and attachment avoidance, as well as the patients’ perceived quality of the working alliance and therapeutic agency in psychotherapy sessions predict their level of COVID-related posttraumatic distress over time. Moreover, we aim to examine potential differences in the utilization of the online therapy process between patients with different levels of attachment anxiety and avoidance. 

Specifically, the study’s research aims are threefold; (1) Examining patients’ attachment anxiety and avoidance, and their self-reported quality of the working alliance and therapeutic agency in online therapy, as well as their level of COVID-related distress at the start of the pandemic and three months later. We expected patients to endorse moderate levels of attachment anxiety and avoidance, comparable to previously reported outpatient populations. In line with previous research [49,57], we hypothesized that the patients’ ratings for working alliance and therapeutic agency in online sessions would be relatively moderate, similar to in-person therapy sessions, as reported by patients in clinical samples pre-COVID [59], and by therapists in other pandemic contexts [60]. We also expected patients to endorse moderate levels of COVID-related traumatic distress, similar to the levels of distress reported in community samples during the pandemic; (2) Examining to what extent of the patients’ attachment, working alliance, and therapeutic agency predict their experienced traumatic distress in the context of the pandemic three months later. Based on studies relating low attachment anxiety, low attachment avoidance, and high working alliance to better psychological functioning [34,49,59,61], we hypothesized that lower attachment anxiety and attachment avoidance, as well as higher working alliance and therapeutic agency in their online treatment would predict lower levels of COVID-related traumatic distress at follow-up, once controlled for baseline traumatic distress; (3) Examine if the patients’ experiences of the collaborative processes in therapy (i.e., a strong working alliance and high levels of therapeutic agency) have a differential impact on COVID-related traumatic distress at follow-up among patients with different levels of attachment anxiety and avoidance. Based on the high interpersonal dependency needs of people who report attachment anxiety, we hypothesized that for the patients who are more anxiously attached the relationship between the collaborative process variables (working alliance and therapeutic agency) and COVID-related traumatic distress would be stronger than for those patients who are less anxiously attached or avoidantly attached [48,62].

## 2. Materials and Methods

### 2.1. Sample

Psychotherapy patients, who had been in therapy before the onset of the pandemic, and continued to receive therapy online via videoconferencing during the pandemic were recruited via social media posts (Facebook, Twitter, Reddit), as well as local neighborhood listservs during the widespread lockdowns (April–July 2020; for baseline). The participants who completed this initial survey were invited to participate in a follow-up survey three months later (July–September 2020). In the present study, we included all participants who completed the relevant measures at baseline (*N* = 466), which included the subgroup of 121 participants who also completed measures at follow-up.

### 2.2. Measures

Demographic Variables. A number of demographic questions were included in the survey, such as age, gender, ethnicity, location, employment, education, relationship status, mental health diagnoses, setting of sessions before the pandemic, and number of received therapy sessions. 

Experiences in Close Relationship Questionnaire-Revised Short-form (ECR-RS) [19] was used to assess attachment. The ECR-RS is a 9-item version of the 36-item Experiences in Close Relationships Questionnaire—Revised (ECR-R). The ECR-RS is a self-report measure designed to assess attachment patterns in close relationships and can be used to assess relationship-specific attachment with specific individuals, and general attachment. In the present study, participants complete the measure regarding how they feel in close relationships in general. Items are scored on a 7-point Likert scale (1—*Strongly disagree*; 7—*Strongly agree*). Two scores can be computed; attachment-related avoidance (e.g., “I don’t feel comfortable opening up to others”) and attachment-related anxiety (e.g., “I’m afraid that other people may abandon me”). The ECR-RS has been found to be a reliable and valid measure and its scores are relatively stable over time [19]. Based on a ECR-RS study conducted in a large community sample reported by Fraley and colleagues (2011), the average attachment anxiety in the general population can be expected to be around 2.53 (*SD* = 1.19), whereas the average attachment avoidance is expected to be 3.18 (*SD* = 0.96). In a small outpatient sample at a university clinic [63] the average attachment anxiety on the ECR-RS was reported as 3.79 (*SD* = 1.13) and the attachment avoidance was 3.29 (*SD* = 1.22). In our study the internal consistency of the attachment avoidance and attachment anxiety scales were 0.94 and 0.90, respectively.

The Working Alliance Inventory-Short Form Revised (WAI-SR) [59,64] is a 12-item patient self-report scale that measures three domains of the therapeutic alliance: agreement between patient and therapist on the goals of the treatment (Goal; e.g., “The therapist and I are working towards mutually agreed upon goals”.; agreement between patient and therapist about the tasks to achieve these goals (Task; e.g., “I believe the way we are working with my problem is correct”); and the quality of the bond between the patient and therapist (Bond; e.g., “I believe my therapist likes me’’). Each item is rated on a 5-point Likert scale anchored at each end with ‘rarely or never’ (1) and ‘always’ (5). In line with more recent recommendations [65], the overall mean WAI-SR score, rather than subscale scores, was used in this study. Higher scores indicate a better working alliance. The WAI-SR has high internal consistency (Cronbach’s alpha of the total score is 0.9; [59,64] and good construct validity as indicated by associations with other alliance measures and by prediction of therapy outcome [59,61]. In a psychometric study on the WAI-SR [59], the average WAI-SR score for in-person outpatient therapy in their sample of 88 patients was reported as 3.8 (*SD* = 0.63). More recently, in a previous study on a 20-session treatment of an in-person outpatient sample (*N*= 386), in which therapeutic agency was also assessed, the average WAI-SR ranged from 3.65 to 3.88 (*SD* ranging from 0.70–0.77) depending on the phase of treatment [56]. Although no studies reporting on the patient-reported WAI-SR in online therapy during COVID appear to have been published, therapist-reported WAI-SRT scores during online therapy during COVID suggest similar ratings of the working alliance (*M* = 4.09, *SD* = 0.48; [60]. In our study we phrased the instruction in line with the unique context: “Since the pandemic, during your online sessions….” The Chronbach’s alpha of the total score in our sample was 0.66. 

Therapeutic Agency Inventory (TAI; [49]). The TAI is a newly developed 15-item patient self-report scale that has shown to be reliable, valid, and change-sensitive, and that can be used to assess patients’ sense of agency in psychotherapy [49,57]. The items are answered on a 5-point Likert scale ranging from 1 (not true) to 5 (very true), with a higher TAI score reflecting a higher sense of agency. Example items include: “I try out new things in between sessions”, “If I don’t like something about my therapy, I address my concerns with my therapist”, “I take an active part in determining the course of my therapy”. Internal consistency has shown to be high (e.g., [56]) and its validity is supported by positive associations with general self-efficacy expectations, control expectations in psychotherapy, common change mechanisms (e.g., alliance, mastery), and negative correlations with psychological distress [57]. In a previous study on a 20-session treatment of an in-person outpatient sample (*N* = 386), the average TAI ranged from 3.66 to 3.81 (*SD* ranging from 0.52–0.55) depending on the phase of treatment. For purposes of our study we phrased the introduction of this measure as: “How do these statements reflect your online therapy sessions since the pandemic?” In our study, Cronbach’s alpha was 0.83.

Impact of Event Scale—Revised (IES-R; [66]). The IES-R is a 22-item self-report measure that assesses subjective distress caused by traumatic events. Respondents are asked to identify a specific stressful life event and then indicate how much they were distressed or bothered during the past seven days by each “difficulty” listed. Items are rated on a 5-point scale ranging from 0 (“not at all”) to 4 (“extremely”) and include, for example: “I felt as if it hadn’t happened or wasn’t real.”, “I was jumpy and easily startled.”, “I tried not to think about it.” The IES-R yields a total score (ranging from 0 to 88), which is used as a measure of severity of post-traumatic stress symptoms (PTSS), rather than as a tool to diagnose post-traumatic stress disorder (PTSD). Following protocols used in numerous studies during pandemics (e.g., [5]), the instruction’s wording was modified to reflect the COVID-19 pandemic as the identified stressor. For example: “For the past week, how much have you been distressed or bothered by the following difficulties related to the coronavirus/COVID-19 pandemic?” In a large mainly American community sample (*N* = 725) surveyed during the very first weeks of the COVID outbreak the average level of traumatic distress was 28.01 (*SD* = 18.04), which is higher than levels reported in several small outpatient clinical samples in Italy (e.g., *M* = 18.15; *SD* = 13.67; *N* =110, see [67] and in China (e.g., *M* = 17.7; *SD* = 14.2; *N* = 76, see [68]). The Cronbach alpha in this study was 0.81 at baseline and 0.82. at follow-up.

### 2.3. Statistical Analyses

Analyses were performed with SPSS, version 25. Descriptive data were used to characterize the sample and study the frequency distribution of the variables of interest. Preliminary tests were conducted to assess normality of the data and associations with demographic variables. All statistical tests were two-tailed, with alpha set at 0.05. 

To examine our first research question, descriptive data were reported and compared with available benchmarks from previous publications using these same measures. To test our hypotheses regarding whether patients attachment anxiety and attachment avoidance, working alliance, and therapeutic agency predicted COVID-related traumatic distress at the 3-month follow-up, we conducted multiple linear regression analyses and controlled for the effects of COVID-related traumatic distress at baseline. To assess whether patients’ attachment anxiety and avoidance impact the importance of the collaborative therapy experience variables (working alliance, therapeutic agency) in predicting COVID-related traumatic distress symptoms three months later, we built four moderation models. Each model included the scores of one attachment variable (anxiety/avoidance), one therapy process variable (working alliance/therapeutic agency), and the relevant interaction term (attachment anxiety/avoidance × working alliance/therapeutic agency) to predict COVID-related traumatic distress three months later, while controlling for baseline COVID-related traumatic distress. Conditioning values (−SD, Mean, +SD) and Johnson-Neyman output were used to identify the relevant cutoff levels of the moderators. 

## 3. Results

### 3.1. Description of Sample Characteristics

The 466 patients were on average 30.61 years old (*SD* = 19.05, range: 18–78). The majority of patients were White (*n* = 393; 84.3%), female (*n* = 354; 76%), single (*n* = 258; 55.4%), and from the United States (*n* = 364; 78.1%). In line with the inclusion criteria of the survey, all patients reported that they had attended therapy sessions before the pandemic (94.8% in in-person therapy, 2.4% in phone therapy, and 2.8% in video conferencing therapy). The majority (*n* = 411, 88.2%) reported at least one mental health diagnosis before the start of the pandemic. For a more detailed demographic description of the sample, please see Table 1.

The mean and standard deviation of each of the variables (attachment avoidance, attachment anxiety, working alliance, therapeutic agency, and COVID-related traumatic distress at the two timepoints) are provided in Table 2. Patients in our study reported a level of attachment anxiety (*M* = 3.00, *SD* = 1.83) that is significantly higher (*t* = 2.71, *df* = 116, *p* < 0.01) than the estimation in the general population (*M* = 2.53 in Fraley et al., 2011 [19]) but significantly lower than the value reported in a small university clinic outpatient sample (*t* = 2.88, df = 167, *p* < 0.01; *M* = 3.79, *SD* = 1.13 in [63]. In our sample, patients’ level of attachment avoidance (*M* = 2.74, *SD* = 1.59) was significantly lower than the estimation in the general population (*t* = −3.00, df = 116, *p* < 0.01; *M* = 3.18, *SD* = 0.96) in Fraley et al., 2011 [19]) or in the outpatient sample (*t* = 2.22, df = 167, *p* = 0.03; *M* = 3.29, *SD* = 1.22, in [63].

Overall, patients reported having a relatively good working alliance with their therapists (*M* = 3.75, *SD* = 0.84; range: 1.00–5.00), on par with patient WAI-SR alliance ratings reported in the few available studies on in-person patient samples (e.g., [49,59]. Similarly, their reported therapeutic agency (*M* = 3.68, *SD* = 0.57) was similar to previously reported levels [56].

Regarding COVID-related post-traumatic distress, patients reported an average level of mild distress both for baseline (*M* = 11.97, *SD* = 5.45) and 3-month follow-up (*M* = 10.59, *SD* = 5.28) with a slight decrease over time, which on average did not reach the level of statistical significance (*t* = 0.87, df = 120, *p* = 0.39). These levels of COVID-related traumatic distress were significantly lower than the distress reported in a large American community sample (*M* = 17.54; *SD* = 16.17 for males and *M* = 26.47; *SD* = 16.80 for females in [5]; *p* < 0.001 for all comparisons). Gender, age, education, or time of survey completion at follow-up were not related to COVID-related traumatic distress, (*p* > 0.05; see Supplement). However, COVID-related traumatic distress symptoms at baseline differed depending on the time when patients completed the baseline survey (*F* (6, 459) = 2.48, *p* = 0.02), and post-hoc analysis indicated that patients who completed the baseline questionnaires in June reported significantly higher traumatic distress (*N* = 225, *M* = 12.55, *SD* = 5.35) than patients who completed the questionnaires in April (*N* = 31, *M* = 8.93, *SD* = 4.98; *p* = 0.01). Thus, the month of data completion at baseline and COVID-related traumatic distress at baseline are used as a covariate in further analyses.

### 3.2. Predictions of COVID-Related Post-Traumatic Distress

The Pearson correlations among these variables suggests that attachment avoidance was negatively related with working alliance and therapeutic agency, while being positively related with attachment anxiety (Table 2). Working alliance and therapeutic agency were positively related. COVID-related traumatic distress at baseline was negatively related to therapeutic agency. Collinearity diagnostics across the regression analyses suggested that there was no concern about multicollinearity, as variance inflation factors (all <1.80) were well below the commonly used threshold of 10, and tolerance values (all > 0.50) were well above commonly used threshold of 0.10 [69]. 

Stepwise multiple regression indicated that the level of COVID-related traumatic distress at follow-up was predicted by COVID-related traumatic distress at baseline (*p* < 0.001) and the time when participants completed baseline questionnaires (*p* = 0.03) in the baseline model, which explained 40.7% of the variance. After controlling these covariates, COVID-related traumatic distress at follow-up was further predicted by attachment anxiety (*B* = 0.20, *SE* = 0.08, *p* < 0.01) but not attachment avoidance, therapeutic agency, or working alliance. This model explained 45% of variance in traumatic distress at follow-up (*F*(6, 110) = 15.00, *p* < 0.001). See Table 3 for detailed regression results. 

**Figure 1:**
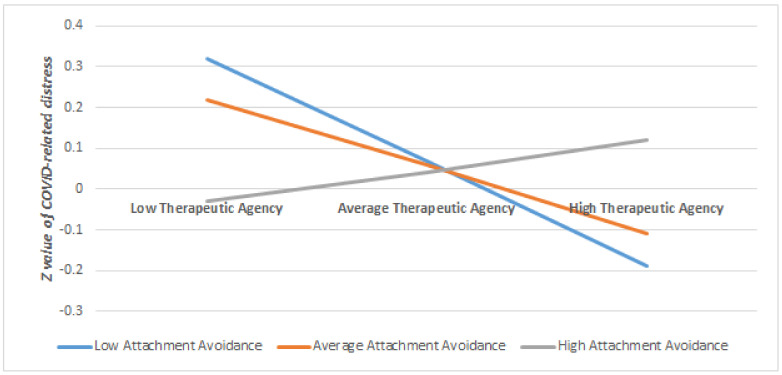
The Moderating Role of Attachment Avoidance on the Relationship between Therapeutic Agency and COVID-related Traumatic Distress at Follow-up, once Controlled for Baseline Distress and Time Passed.

Regarding the moderation effects on the outcome of COVID-related traumatic distress, we found significant moderation effects of attachment anxiety on working alliance (*B* = −0.17, *p* = 0.01), indicating that when patients had higher levels of attachment anxiety (>0.72 *SD*), higher working alliance predicted lower COVID-related traumatic distress at follow-up after controlling for covariates (see Table 4). When the level of attachment anxiety is lower (<0.72 *SD*), the working alliance did not significantly predict COVID-related traumatic distress at follow-up. In other words, stronger working alliance was significantly associated with lower COVID-related traumatic distress at follow-up only for those who have a higher level of attachment anxiety (for a visual illustration of this moderation see Figure 1). 

**Figure 2:**
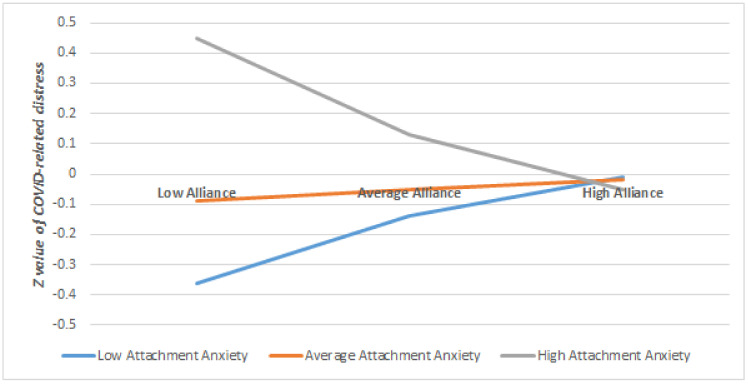
The Moderating Role of Attachment Anxiety on the Relationship between Working Alliance and COVID-related Traumatic Distress at Follow-up, once Controlled for Baseline Distress and Time Passed.

Furthermore, when patients scored low on attachment avoidance (<−0.40 *SD*), higher therapeutic agency was related to lower levels of COVID-related traumatic distress at follow-up, after controlling for the covariates. If the patients scored higher than −0.40 *SD* on attachment avoidance, the therapeutic agency did not significantly relate to COVID-related distress at follow-up (Table 4). In other words, higher levels of therapeutic agency was significantly associated with lower levels of COVID-related traumatic distress only if the patients scored low on attachment avoidance (for a visual illustration of this moderation see Figure 2). There was no significant moderation of attachment anxiety on the relationship between therapeutic agency and COVID-related traumatic distress, nor for attachment avoidance on working alliance and COVID-related distress (see Supplementary Materials Table S1). 

## 4. Discussion

The COVID-19 pandemic created a uniquely challenging situation for psychotherapy patients with pre-existing mental health conditions. In the present study, we posited that patients’ attachment anxiety and attachment avoidance, and patients’ experiences of the collaborative processes (as measured by the working alliance and therapeutic agency) in online therapy during the pandemic might be relevant to and predictive of patients’ traumatic distress over time. We further hypothesized that patients’ attachment anxiety and attachment avoidance might moderate the relationship between the working alliance and therapeutic agency on COVID-related traumatic distress at the three month follow-up. 

In answer to our first hypothesis, we found that the level of attachment avoidance in our sample was lower than community (e.g., [19]) and clinical populations (e.g., [63]), which might be explained by the lower likelihood of these avoidant patients participating in an online survey posted on social media. Patients in our sample reported a relatively good quality of their working relationship with their therapist in their online sessions, and reported moderate levels of therapeutic agency, both in line with publications on in-person therapies pre-COVID-19 (e.g., [49,59]). The survey responses of our sample of online patients also confirmed that their perception of the quality of the working alliance and therapeutic agency in their online therapy was similar to those reported by in-person patients previously. More specifically, the majority of the 466 patients who participated in the survey reported higher levels of attachment anxiety than the general population (e.g., [19]), indicating relatively insecure attachment representations. Our patient sample reported relatively mild symptoms of COVID-related post traumatic distress at both timepoints, compared to published data from a large community sample, of which probably most were not currently in psychotherapy. Notably, the levels of COVID-related traumatic distress differed depending on the month in which the initial survey was completed, suggesting that patients reported higher levels of traumatic stress in June than in April 2020. The subsample of 121 patients who also completed a follow-up survey, reported low levels of improvement over a 3 months’ time, not reaching levels of statistical or clinical significance. Although this might imply that patients overall struggled to adapt to the pandemic situation and/or that treatment was not effective in reducing the COVID-related traumatic distress, the significant covariate of time of survey completion suggests that the pandemic-related impact on patients’ lives intensified during the Spring of 2021 and that patients (together with their therapists) could have done relatively well in managing to keep their COVID-related traumatic distress levels relatively constant. 

The results partly confirmed our second hypothesis about the prediction of patients’ resilience. We found that lower levels of COVID-related traumatic distress at the three month follow-up was predicted by patients’ low levels of attachment anxiety and high levels of therapeutic agency in their online sessions, while controlling for baseline distress. Notably, attachment avoidance and working alliance did not significantly contribute to the prediction of traumatic distress. This suggests that patients with higher attachment anxiety and relative lack of agency in their online treatment are more vulnerable to maintain distress levels over time during the pandemic compared to other patients, and vice versa: patients with less attachment anxiety and more agency displayed more resilience over time.

On the other hand, patients’ level of attachment avoidance and their perception of the online working alliance might be less prominent in predicting traumatic distress over time in patients receiving online therapy than in in-person sessions [40].

The significance of attachment anxiety as opposed to attachment avoidance to directly and negatively predicting COVID-related traumatic distress over time is important to consider. There is accumulating evidence that attachment avoidance is associated with higher functioning and better outcomes in clinical samples compared to attachment anxiety [32,37,38], and our study also indicates that patients with higher attachment anxiety also have higher levels of distress compared to other patients over time. 

Our finding that patients’ level of attachment avoidance does not predict COVID-related traumatic distress over time, appears to fit the results from a previous meta-analysis on adult attachment and PTSD symptoms, in which they concluded that attachment avoidance is not related to symptoms of PTSD, whereas high levels of attachment anxiety were strongly related to PTSD symptoms [34]. Woodhouse and colleagues (2015) suggested that avoidance of trauma-triggers, combined with avoidance of attachment related worries, may help patients who have experienced a traumatic event [36], whereas anxiety around emotional dependency might intensify the traumatic distress. 

Therapeutic agency, which was the other significant predictor of patients’ resilience over time, has been proposed to be a common factor of change in psychotherapy [70,71] and our results support its importance in the specific context of the pandemic and online psychotherapy. It is noteworthy that the working alliance was not a direct predictor of COVID-related traumatic distress over time across the sample. This goes against the psychotherapy literature on working alliance and outcome in in-person therapy [40], and thus might be explained by the remote therapy format, where relational processes may play out slightly differently. Even though there is accumulating evidence showing that the level of therapeutic alliance is comparable in in-person and online settings [43], there is much less evidence regarding the possibly differential impact of therapeutic alliance on therapy outcome in the online therapy process.

Finally, our third hypothesis was also partially confirmed. Working alliance and therapeutic agency, both conceptualized as core concepts in the literature on psychotherapy change process [40,49,70], may indeed play a different role in the therapy of patients with more or less attachment anxiety or attachment avoidance. More specifically, patients’ high attachment anxiety moderated the relationship between high working alliance and COVID-related traumatic distress three months later, which indicates that those patients with strong need for approval and concerns about being rejected or abandoned by others, a high-quality working alliance with their therapist may be crucial in order to be able to adapt to the traumatic distress during the pandemic [62]. They may especially benefit from a new, positive attachment experience with the therapist that will allow them to experience the therapeutic relationship as a secure base [13] from where they can start working on themselves and experience a relief of distress. Despite the physical and emotional distance in remote sessions [72], these patients’ insecurities and fear of rejection and abandonment might have been triggered by the pandemic, resulting in relatively high scores on the working alliance and therapeutic agency scales. There is preliminary evidence the therapeutic relationship has shifted due to the shared stress of the pandemic [73], which means that for patients high on attachment anxiety, having a continued relationship with the therapist might have been even more important now than in pre-pandemic times (hence the higher alliance scores). For patients who experience less attachment anxiety (either those who are more attachment avoidant or more securely attached), a highly rated working alliance might be less crucial in order to achieve improvement. 

Contrary to the moderating role of attachment anxiety, attachment avoidance appears to play a different role. Patients with high attachment avoidance might rely less on the collaborative process with the therapist, as measured by the self-reported working alliance and therapeutic agency in their online sessions. This may imply that those patients may relate to their therapists in a similarly avoidant, emotionally detached way [74], and may be more reluctant to emotionally engage in the therapy process, not wanting to become dependent on the therapist (or the therapy), which might show as relatively lower levels of therapeutic agency. All in all, this supports the notion that individual differences in early relationships with the primary caregivers are carried forward and shape relationships with others in adulthood, including one’s therapist [13,15,16,17,18]. In fact, some argue that John Bowlby developed his original theory of attachment partly to explain why some of his patients appeared to avoid intimacy and defend against experiencing emotions, even though it had disastrous consequences for their social adaptation [75]. 

For patients on the low end of the attachment avoidance continuum (i.e., who were relatively more securely attached) higher therapeutic agency in their online sessions predicted low levels of COVID-related traumatic distress over time. This suggests that those who are able to be healthily engaged and dependent on others in their interpersonal relationships (i.e., low on attachment avoidance) and also to be more engaged, cooperative and more able and willing to take charge of their therapeutic work (agency), benefit from it the most compared to other patients, in terms of experiencing less distress over time. These low-avoidant patients may be able to best use their sense of agency in therapy to achieve lower rates of traumatic distress at the three month follow-up. Notably, based on the results of the multiple regression analysis, all patients (even patients who are higher on attachment avoidance) would benefit from developing higher levels of therapeutic agency in their therapies in order to increase their resilience to experience distress over time. 

## 5. Limitations

Several important study limitations can be considered: First, it remains unclear how generalizable our findings are to psychotherapy patients worldwide in online and in-person treatments, in and outside COVID-19. Our study reports on the largest clinical sample yet of psychotherapy patients’ perceived quality of the online working alliance and therapeutic agency. The use of social media for patient recruitment resulted in a relatively heterogeneous patient sample with regards to geographical location, length of current treatment, and mental health diagnosis [76]. However, our patient sample was biased towards a middle to high socioeconomic status. The majority of patients were white, well-educated women who were employed and single, who could afford longer-term private psychotherapy. Notably, in our follow-up sample, only eight percent identified as male. Also, the age range in our sample was very wide. Older patients may have found the transition to teleconferencing more challenging than younger patients, because of differences in familiarity with electronic devices and communication. Besides these patient characteristics, the unique timing of the data-collection potentially also limits its generalizability. On the one hand, our study had a broad scope relevant when examining the effects of a global pandemic [77]. On the other hand, the timing of our study was unique in that we collected data during a period of intense stress and uncertainty during the first three months of the pandemic, after patients’ suddenly switched to online therapy. It currently remains unclear if the patients’ attachment, working alliance and therapeutic agency would have played the same role in patients’ resilience if this had been assessed in online sessions outside of the pandemic, or in in-person sessions during the pandemic. 

Notably, our comparisons with benchmarks of the working alliance and therapeutic agency measures used in in-person therapy pre-pandemic should be interpreted very tentatively, because the phrasing of the instructions was adapted for this study. Similarly, the outcome measure used in this study was a self-report scale of traumatic distress, especially adapted for the COVID context. Although this adaptation of the items was used in many other COVID-related studies (e.g., [5]), it is important to note that these symptoms might not reflect the patients’ treatment goals or outcomes per se, especially outside of the pandemic context. In other words, it is possible that their COVID-related traumatic distress are not reflective of the patients’ treatment outcomes per se or that their attachment anxiety and therapeutic agency do not predict their symptoms targeted in treatment. That said, it might be argued that online therapy was nevertheless effective for these patients, in offering collaborative support to keep post-traumatic stress at bay.

Moreover, for purposes of this study the survey was kept relatively short (15 min) in order to limit the burden on the patient participants. However, within this pandemic context, several societal and personal variables could have been worthwhile to consider, including patients’ access to national and local mental health services, the exact timing and implementation of COVID-related governmental policies, as well as their own physical health, and financial situation before and during the pandemic. Also, given that increases in therapeutic agency over the course of psychotherapy have shown to predict subsequent symptom improvement [56], it would have been relevant to examine therapeutic agency repeatedly at several time points in treatment. In addition, our patient sample might be reflecting many more individual differences that were not assessed in this survey study, and should be considered in future research. For example, patients might have received therapy for a range of reasons, experienced different types of intrapsychic or interpersonal stress symptoms, been in therapy for different lengths of time and been receiving various types and formats of psychotherapy before the sudden transition to online therapy. 

Lastly, given the continuation of the COVID-19 pandemic and its societal and personal consequences long beyond the three months assessed in this study, it would have been informative to have included repeated follow-up measurements to track these patients’ traumatic distress longer-term. It is possible that the patients’ attachment anxiety and avoidance, as well as their perceived working alliance, and therapeutic agency play a different role in patients’ resilience at follow-up time periods of 6 or 12 or 18 months. 

## 6. Conclusions

Given that our study was conducted under unique circumstances, the ongoing pandemic and forced transition to online therapy sessions might have impacted our results in ways unbeknown to us yet. Within the context of these limitations, the study suggests that the assessment of patients’ attachment representations, and their perceived quality of the working alliance and therapeutic agency might be important to consider in (online) psychotherapy. Patients have different ways in which they can make use of the therapeutic process, based on their own early attachment experiences, and this should be emphatically explored to avoid stagnation of therapy progress or possibly treatment drop-out. The study findings support the differential impact of attachment anxiety and avoidance on reducing traumatic distress, as well as the potential relevance of patients’ sense of agency in the therapy process. More research on understanding how attachment relates to the interaction between patients and therapists, and subsequent symptom relief, as well as the potential confounding role of the pandemic and/or the online therapy format seems warranted, and might invite slightly different relational and agential processes [73] (Békés, Aafjes-van Doorn, & Roberts, 2021). Nevertheless, even though the pandemic-related stressors will hopefully diminish over time, the online therapy format appears to be here to stay, and studies aiming to understand processes specific to online sessions are thus warranted. Future research should explore generalizability of our findings both in in-person and online settings outside of the stresses of the current pandemic. 

## Figures and Tables

**Table 1:** Patients’ Characteristics (*N* = 466).

Variable	*N* (%)	
	Baseline	Follow-Up
	(*N* = 466)	(*N* = 121)
**Gender**		
Female	354 (76.0)	102 (84.3)
Male	94 (20.2)	10 (8.3)
Nonbinary	18 (3.9)	9 (7.4)
**Ethnicity**		
White	393 (84.3)	107 (88.4)
Asian/Asian Indian/Pacific Islander	40 (8.6)	12 (9.9)
Hispanic/Latinx/Spanish	21 (4.5)	4 (3.3)
Black/African American	11 (2.4)	2 (1.7)
American Indian/Alaskan Native	10 (2.1)	2 (1.7)
Middle Eastern	5 (1.1)	2 (1.7)
Other	19 (4.0)	10 (8.2)
**Location**		
USA	364 (78.1)	96 (79.3)
Europe	34 (7.3)	9 (7.4)
United Kingdom	17 (3.6)	7 (5.8)
Canada	11 (2.4)	4 (3.3)
Australia	10 (2.1)	2 (1.7)
India	14 (3.0)	2 (1.7)
Other	16 (3.4)	0
**Employment**		
Employed full time	228 (48.9)	50 (41.3)
Employed part time	81 (17.4)	26 (21.5)
Student	132 (28.3)	39 (32.2)
Unemployed/looking for work	47 (10.1)	10 (8.3)
Disabled	24 (5.2)	7 (5.8)
Retired	4 (0.9)	2 (1.7)
Other	19 (4.1)	5 (4.1)
**Education**		
Less than high school or high school	22 (4.7)	3 (2.5)
Professional degree (e.g., trade school)	28 (6.0)	8 (6.6)
Some college	104 (22.3)	23 (19.0)
College	164 (35.2)	47 (38.8)
Master’s degree	126 (27.0)	32 (26.4)
Doctorate	22 (4.7)	8 (6.6)
**Relationship status**		
Married/cohabiting	184 (39.5)	39 (32.2)
Single/never married	258 (55.4)	79 (65.3)
Widowed/divorced/separated	21 (4.5)	3 (2.5)
**Mental health diagnosis** *		
Depression	274 (58.8)	73 (60.3)
PTSD	121 (26.0)	34 (28.1)
Anxiety	311 (66.7)	85 (70.2)
Bipolar	37 (7.9)	8 (6.6)
Attention Deficit Hyperactivity Disorder	94 (20.2)	22 (18.2)
Obsessive-Compulsive Disorder	42 (9.0)	10 (8.3)
Eating Disorder	73 (15.7)	20 (16.5)
Personality Disorder	48 (10.3)	10 (8.3)
Autism Spectrum Disorder	25 (5.4)	7 (5.8)
Substance-Use Disorder	21 (4.5)	5 (4.1)
Other	41 (8.8)	13 (10.7)
No diagnosis	55 (11.8)	18 (14.9)
**Setting of therapy before the pandemic** *		
Private practice	363 (77.9)	100 (82.6)
Outpatient clinic	62 (13.3)	13 (10.7)
Hospital	44 (9.4)	2 (1.7)
Inpatient clinic	26 (5.6)	0 (0)
Online/by phone	31 (6.7)	6 (5.0)
Other	25 (5.4)	10 (8.3)
**Time of completion baseline survey**		
April	31 (6.9)	10 (8.3)
May	145 (32.1)	55 (45.5)
June	225 (49.8)	46 (38.0)
July	51 (11.3)	10 (8.3)
**Number of sessions with current therapist before the pandemic**		
Less than 5	69 (14.8)	6 (5.0)
10–5	68 (14.6)	12 (9.9)
19–10	67 (14.4)	15 (12.4)
20 or more	246 (52.8)	83 (68.6)
None, just started	15 (3.2)	5 (4.1)

Notes. * Multiple options were possible to select.

**Table 2:** Means (Standard Deviations) of Study Variables and Pearson Correlations Between Standardized Variables.

Variables	*N*	Mean (SD)	1	2	3	4	5.	6
1. ECR-RS Avoidance	117	2.74 (1.59)	--					
2. ECR-RS Anxiety	117	3.00 (1.83)	0.28 **	--				
3. WAI-SR	466	3.75 (0.84)	−0.43 **	−0.06	--			
4. TAI	466	3.68 (0.57)	−0.22 *	0.10	0.57 **	--		
5. IES-R at baseline	466	11.97 (5.45)	0.01	−0.01	0.05	−0.13 *	--	
6. IES-R at follow-up	121	10.59 (5.28)	−0.04	0.14	0.01	−0.13	0.62 **	--

Notes. ECR-RS = Experiences in Close Relationship Questionnaire-Revised Short-form; TAI = Therapeutic Alliance Inventory; WAI-SR = Working Alliance Inventory Short-form Revised; IES-R = Impact of Event Scale—Revised; SD = Standard Deviation. * *p* < 0.05; ** *p* < 0.01.

**Table 3:** Standardized Regression Coefficients for Multiple Linear Regression Models for IES-RS at Follow-up.

Direct Effects	Estimate	*SE*	*t*	*p*
**Model 1**				
IES-R at baseline	0.63	0.07	8.50	<0.001 ***
Time of completion	0.21	0.10	2.15	0.03 *
**Model 2**				
IES-R at baseline	0.64	0.07	8.70	<0.001 ***
Time of completion	0.15	0.11	1.44	0.15
ECR-RS Anxiety	0.20	0.08	2.59	0.01 *
ECR-RS Avoidance	−0.11	0.08	−1.25	0.21
TAI	−0.13	0.09	−1.34	0.17
WAI-SR	−0.01	0.10	−0.14	0.89

*Notes.* ECR-RS = Experiences in Close Relationship questionnaire-Revised Short-form; TAI = Therapeutic Agency Inventory; WAI-SR = Working Alliance Inventory Short-form Revised; IES-RS = Impact of Event Scale—Revised; Estimate = Unstandardized coefficient using standardized variables; SE = Standard Error. * *p* < 0.05; *** *p* < 0.001.

**Table 4:** Results and Conditional Effects of Significant Moderations of Attachment on the Relationship between the Collaborative Therapy Variables and COVID-related Traumatic Distress at Follow-up, once Controlled for baseline COVID-related Distress and Completion Time.

Attachment Anxiety	Coefficient	SE	*t*	*p*	LLCI	ULCI
ECR-RS Anxiety * WAI-SR						
ECR-RS Anxiety	0.17	0.07	2.48	0.01 *	0.04	0.31
WAI-SR	−0.03	0.06	−0.47	0.64	−0.17	0.10
Moderation: ECR-RS Anxiety X WAI-SR	−0.17	0.07	−2.63	0.01 *	−0.31	−0.04
Covariate: time of completion	0.23	0.09	2.47	0.02 *	0.49	0.77
Covariate: IES_T0	0.62	0.07	8.80	0.000 ***	0.49	0.77
*Conditional Effects* *Value of the Moderator*						
ECR-RS Anxiety	WAI-SR					
−1 SD	0.16	0.11	1.51	0.13	−0.05	0.37
0 SD	−0.04	0.07	−0.63	0.53	−0.18	0.09
0.72 SD (cutoff)	−0.16	0.08	−1.98	0.05 *	−0.32	0.00
+1 SD	−0.22	0.09	−2.31	0.02 *	−0.40	−0.03
**Attachment Avoidance**						
ECR-RS Avoidance * TAI						
ECR-RS Avoidance	−0.07	0.08	−0.85	0.40	−0.22	0.09
TAI	−0.10	0.08	−1.26	0.21	−0.25	0.06
Moderation: ECR-RS Avoidance X X TAI	0.17	0.08	2.15	0.03 *	0.01	0.32
Covariate: time of completion	0.14	0.10	1.38	0.17	−0.06	0.35
Covariate: IES_T0	0.61	0.07	8.39	0.000 ***	0.47	0.76
*Conditional Effects* *Value of the Moderator*						
ECR-RS Avoidance	TAI					
−1 SD	−0.27	0.11	−2.47	0.02 *	−0.50	−0.06
−0.40 SD (cutoff)	−0.16	0.08	−1.98	0.05 *	−0.32	0.00
0 SD	−0.09	0.08	−1.18	0.24	−0.24	0.06
+1 SD	0.07	0.11	0.60	0.55	−0.15	0.28

Notes. The dependent variable is COVID-related traumatic distress at follow-up, measured by the IES-R. The covariates are COVID-related traumatic distress at baseline, measured by the IES-R (IES-T0), and the time of completion, reflecting the month. Only significant moderation results were presented in this table (non-significant results are presented in the Supplementary Materials Table S1). All the variables are standardized before adding to the models. ECR-RS = Experiences in Close Relationship questionnaire-Revised Short-form; TAI = Therapeutic Agency Inventory; WAI-SR = Working Alliance Inventory Short-form Revised; IES-R = Impact of Event Scale—Revised; SE = Standard Error; CI = Confidence interval; *LL* = lower limit; *UL* = upper limit. * *p* < 0.05; *** *p* < 0.001.

## Data Availability

Data are available upon request from the corresponding author. The data are not publicly available due to follow-up measurements still in progress.

## Supplementary Materials

The following are available online at https://www.mdpi.com/article/10.3390/brainsci11101288/s1, Table S1 Non-significant Covariate analyses, Non-significant moderation results.
