# Supplementary material for: COVID-19 Related Traumatic Distress in Psychotherapy Patients during the Pandemic: The Role of Attachment, Working Alliance, and Therapeutic Agency
Source: Brain Sci. 2021 Sep 28;11(10):1288. doi: 10.3390/brainsci11101288 (PMC8533688; doi:10.3390/brainsci11101288)
Supplement: Supplementary file 1 [file brainsci-11-01288-s001.zip › brainsci-1334150-supplementary.pdf]

Supplement

Non-significant Covariates

The Chi-Square test was used to examine whether COVID-related distress differs across patients with different gender or education. Pearson correlations were used to examine whether age was related with COVID-related distress. One-Way ANOVA was used to examine the difference in traumatic stress across time of completion. The results indicated that neither gender or education was related to COVID-related distress (Gender: Chi-Square = 44.55, df = 48, p = .62 for baseline; Chi-Square = 53.29, df = 44, p =.16 for follow-up; Education: Chi-Square = 126.93, df = 144, p =.84 for baseline; Chi-Square = 119.33, df =110, p =.26 for follow-up). Age was not related to traumatic distress either (r = .002, p = .97 for baseline; r = -.04, p = .71 for follow-up). The COVID-related traumatic distress did not significantly differ depending on the month of completion of the follow-up survey, F (3,117) = 1.75, p =.16.

Non-significant Moderation results

Table S1. Non-significant Moderation Results on COVID-related Distress at Follow-up.

Moderation	Coefficient	SE	*t*	*p*
**ECR-RS Avoidance X WAI-SR**				
ECR-RS Avoidance	-.02	.09	-.25	.81
WAI-SR	-.09	.08	-1.07	.29
Moderation: Avoidance X Alliance	.06	.07	.88	.39
Covariate: time of completion	.21	.10	2.09	.04*
Covariate: IES_T0	.64	.08	8.53	<.001***
**ECR-RS Anxiety X TAI**				
ECR-RS Anxiety	.22	.08	2.91	.004**
TAI	-.11	.07	-1.51	.13
Moderation: ECR-RS Anxiety X TAI	-.12	.07	1.90	.06
Covariate: time of completion	.19	.10	1.95	.05
Covariate: IES_T0	.63	.07	8.80	<.001***

Notes. The dependent variable is COVID-related traumatic distress at follow-up, as measured with the IES-RS. The covariates are COVID-related traumatic distress at baseline (IES-T0) and the time of completing baseline questionnaires. The significant moderations are reported in the main manuscript. ECR-RS = Experiences in Close Relationship questionnaire-Revised Short-form; TAI = Therapeutic Agency Inventory; WAI-SR = Working Alliance Inventory Short-form Revised; IES-R = Impact of Event Scale – Revised;

*$p < .05$; **$p < .01$; ***$p < .001$.
